# Cost-effectiveness of a market-based home fortification of food with micronutrient powder programme in Bangladesh

**DOI:** 10.1017/S1368980020003602

**Published:** 2021-04

**Authors:** Sayem Ahmed, Haribondhu Sarma, Zahid Hasan, Mahfuzur Rahman, Mohammad Wahid Ahmed, Mohammad Ashraful Islam, Eric W Djimeu, Mduduzi NN Mbuya, Tahmeed Ahmed, Jahangir AM Khan

**Affiliations:** 1 Health Systems and Population Studies Division, icddr,b, Dhaka 1212, Bangladesh; 2 Health Economics and Policy Research Group, Department of Learning, Informatics, Management and Ethics (LIME), Karolinska Institutet, Stockholm, Sweden; 3 Liverpool School of Tropical Medicine, Pembroke Place, Liverpool L3 5QA, United Kingdom; 4 Nutrition and Clinical Services Division, icddr,b, Dhaka, Bangladesh; 5 Research School of Population Health, The Australian National University, Acton, Australia; 6 Global Alliance for Improve Nutrition, Dhaka, Bangladesh; 7 Health Economics and Policy Unit, School of Public Health and Community Medicine, Sahlgrenska Academy, University of Gothenburg, Gothenburg, Sweden

**Keywords:** Activity-based cost analysis, Cost-effectiveness analysis, Disability-adjusted life years, Home fortification, Micronutrient, Fe deficiency anaemia, Bangladesh

## Abstract

**Objective:**

We estimated the cost-effectiveness of home fortification with micronutrient powder delivered in a sales-based programme in reducing the prevalence of Fe deficiency anaemia among children 6–59 months in Bangladesh.

**Design:**

Cross-sectional interviews with local and central-level programme staff and document reviews were conducted. Using an activity-based costing approach, we estimated start-up and implementation costs of the programme. The incremental cost per anaemia case averted and disability-adjusted life years (DALY) averted were estimated by comparing the home fortification programme and no intervention scenarios.

**Setting:**

The home fortification programme was implemented in 164 upazilas (sub-districts) in Bangladesh.

**Participants:**

Caregivers of child 6–59 months and BRAC staff members including community health workers were the participants for this study.

**Results:**

The home fortification programme had an estimated total start-up cost of 35·46 million BDT (456 thousand USD) and implementation cost of 1111·63 million BDT (14·12 million USD). The incremental cost per Fe deficiency anaemia case averted and per DALY averted was estimated to be 1749 BDT (22·2 USD) and 12 558 BDT (159·3 USD), respectively. Considering per capita gross domestic product (1516·5 USD) as the cost-effectiveness threshold, the home fortification programme was highly cost-effective. The programme coverage and costs for nutritional counselling of the beneficiary were influential parameters for cost per DALY averted in the one-way sensitivity analysis.

**Conclusions:**

The market-based home fortification programme was a highly cost-effective mechanism for delivering micronutrients to a large number of children in Bangladesh. The policymakers should consider funding and sustaining large-scale sales-based micronutrient home fortification efforts assuming the clear population-level need and potential to benefit persists.

One in every three children worldwide is deficient in one or more key micronutrients, limiting their health, development and survival^(1)^. In developing countries, infants and children are the most vulnerable demographic due to their increased nutritional needs for growth and development^(2)^. Countries in sub-Saharan Africa and South Asia have the highest prevalence and absolute numbers of people with micronutrient deficiencies. Additionally, countries in East Asia, Central Asia, Eastern Europe and Latin America have sizeable populations with a high prevalence of micronutrient deficiencies, and thereby contributing to this burden of malnutrition^(3)^.

Micronutrient deficiencies have long-lasting negative impacts on both cognitive and physical development, learning capabilities and work productivity. Although all age groups are vulnerable to the harmful effects of micronutrient deficiencies, these are particularly damaging and difficult to reverse when they occur during fetal development and early childhood^(4)^. Micronutrient deficiencies in infants and young children increase the risk of acquiring infectious illnesses and of death from diarrhoea, measles, malaria and pneumonia^(5)^. In addition to the effects on morbidity and mortality of infants and children, micronutrient deficiencies can exert a significant burden on families, healthcare services, education systems and economies^(6)^. A study conducted by the World Bank found that the countries where populations suffer from a high prevalence of micronutrient deficiencies can experience economic losses as high as 5 % of their gross domestic product (GDP)^(6)^.

In Bangladesh, the burden of malnutrition is also high. A national survey conducted in 2014 revealed that 36 % of children below 5 years of age were stunted, 14 % were wasted and 33 % were underweight^(7)^. The National Micronutrient Survey in 2013 found that 33·1 % of the pre-school aged children were anaemic^(8)^. Access to adequate nutrition as a basic human right is enshrined in the constitution of the Government of the People’s Republic of Bangladesh; therefore, addressing malnutrition is a national priority. To improve the nutritional status of all citizen including children and adolescent girls, the National Nutrition Policy of the Government of the People’s Republic of Bangladesh was endorsed in October 2015^(9)^ and, subsequently, the country’s continued commitment to combat malnutrition in all its forms has been formulated in the second National Plan of Action for Nutrition 2016–2025 with identified priority strategic actions including strengthening multisectoral programmes and increasing coordination among sectors to ensure improved nutrition^(10)^.

The World Health Organization (WHO) recommends home fortification of foods with multiple micronutrient powders (MNP) to improve micronutrient status and address high prevalence of anaemia among children under 2 years of age^(11,12)^. A number of randomised controlled trials, as summarised in a Cochrane review^(4)^, and a recent meta-analysis suggest that home fortification with MNP to be effective in increasing Hb concentration and in improving Fe status and reducing anaemia^(13,14)^ among children under five.

BRAC – a development organisation, based in Bangladesh, aimed to reduce Fe deficiency anaemia (termed as anaemia) through delivering home fortification with MNP among the children of 6–59 months. BRAC used its large network of volunteer community health workers (CHW) to deliver home fortification with MNP through the Maternal, Infant and Young Child Nutrition (MIYCN) programme. The first phase of the programme (2009–2013) established a partnership between Global Alliance for Improve Nutrition (GAIN), BRAC and Renata Limited to test the feasibility of sales of MNP through the BRAC CHWs’ network^(15)^. Based on that experience, the project was expanded to Phase-II (2013–2018) to deliver MNP called Pushtikona-5 through community-level delivery mechanisms, with the aim of reducing micronutrient deficiencies, specifically Fe deficiency anaemia, among children. More details of the programme have been described elsewhere^(14,16)^.

While health intervention programmes and the outcomes are often documented by many researchers, the costs of delivering such programmes are seldom reported^(17)^. Against this backdrop, the purpose of this study was to estimate the start-up cost, implementation cost and cost-effectiveness of the MIYCN home fortification programme, solely run by BRAC in collaboration with Social Marketing Company (SMC), GAIN and Renata Limited, in Bangladesh. Such an assessment of the cost and cost-effectiveness of a large-scale, sales-based delivery model of home fortification in Bangladesh will inform global and national policy, programming and funding decisions regarding investments in childhood Fe deficiency anaemia reduction.

## Methods

### Study design and setting

We compared the cost and effectiveness of the MIYCN home fortification programme with the no intervention scenario. The cost of the programme was estimated from the perspective of both the programme and the beneficiaries and separated into start-up and implementation phases. Based on data from the programme’s evaluation, we ascertained the effectiveness of the programme in terms of anaemia case averted and disability-adjusted life years (DALY) averted from the programme. Finally, the incremental cost per anaemia case averted and per DALY averted was estimated and compared with the GDP-based cost-effectiveness threshold^(18,19)^. The detailed approach of this cost-effectiveness analysis is described in the following sections.

The home fortification programme was implemented in 164 upazilas (sub-districts) out of 492 upazilas in Bangladesh (33·3 %) during 2014–2018. The programme provided age-appropriate counselling on infant and young child feeding and home fortification to improve the micronutrient status of 6–59 months of aged children in the rural communities of the selected upazilas. BRAC’s CHW (under upazila offices) visited the households of the targeted children to provide this intervention and sell micronutrients. In 2014, the population in the selected upazilas was 43 896 814 (30·03 % of the total population in the country). The total number of children aged 6–59 months reached was 5 358 604 for the full programme period. It should be noted here that the MIYCN home fortification programme was initiated with its implementation in sixty-eight upazilas in the first year and scaled-up to additional 120 upazilas in the second year. The programme implementer BRAC decided to start implementing the programme phase-wise to conduct the start-up activities (e.g. training of staff, office set-up, delivering supplies) efficiently.

### Data collection

Seven purposively selected upazila offices from different administrative divisions were visited from May to July 2016 for cost data collection. Semi-structured interviews were conducted with field staff, programme officials and administrative staff, and additionally, data were collected through the review of key monitoring and administrative documents. Specifically, 144 BRAC staff members and volunteer CHW were interviewed to understand the home fortification programme activities and associated costs. We interviewed all programme and supervisory staff (Branch Managers and Program Organizers/officers) in each upazila. Among the CHW, we interviewed randomly selected three Shaysthya Kormi, three Pushti Kormi, six Shaysthya Shebika and six Pushti Shebika in each upazila except Gobindogonj and Muktagacha upazila. In Gobindogonj and Muktagacha upazila, we interviewed higher number of Shaysthya Kormi and Shaysthya Shebika since Pushti Kormi and Pushti Shebika were not recruited in these upazilas by the programme (see online supplementary material, Supplemental Table 1). Interviews with the programme and supervisory staff were conducted to obtain information on time allocation among all activities in order to calculate staff and transportation costs. For those staff with whom interviews were not possible or practical, time allocation estimates were obtained from supervisors. For validation, information was verified with the head office as well as using monitoring document at upazila offices. The output of MIYCN home fortification programme, i.e. different home fortification activities done by BRAC, was collected from the Monthly Performance Report maintained by the upazila offices.

For the different components of the home fortification programme, activity-specific resource utilisation and their magnitudes were extracted from the review of the expenditure reports, visits to field offices and project management offices. At the national level, several offices, such as finance, procurement and supply, were contacted to assist in validating the cost-related information.

The upazila-wise list of under-five children maintained by BRAC was used to estimate the total number of under-five children enrolled in the base year of the programme. Based on that number and additional national statistics, we estimated the total number of children aged 6–59 months in the programme area during 2014–2018.

A physical map segmented sampling was used following the WHO’s Expanded Programme on Immunisation (EPI)-5 sampling procedure^(20)^ to identify the eligible households (*n* 5237). A detailed sampling strategy is explained in another paper^(16)^. The total number of children was separated into complier (ever taken MNP) and non-compiler groups (never taken MNP) based on the MNP coverage estimates from the endline survey (detailed in Effect estimation section). The compiler group was considered as programme beneficiary in this study. The prevalence of anaemia was estimated by measuring the level of Hb of the sampled child. A child was considered as anaemic if the child’s Hb level was <11·0 g/dl. Further, the severe, moderate and mild anaemia were estimated considering the Hb <7 g/dl, Hb = 7–9·99 g/dl and Hb = 10–10·99 g/dl, respectively^(21)^.

#### Cost estimation

An activity-based ingredient costing approach was used to calculate the total economic cost of home fortification programme, including financial costs (accounting costs) and opportunity costs, i.e. the cost of services provided by voluntary staff (e.g. BRAC’s SS). In this approach, all the activities needed to deliver a programme component were identified and then broken down into their inputs or ‘ingredients’, and then costs were estimated for each ingredient^(22,23)^. The activities and their ingredients were identified in consultation with key personnel from BRAC, SMC and GAIN. Further, we consulted BRAC staff members at the upazila level, such as the *Upazila Manager (UM), Programme Organizer (PO), Shasthya Kormi (SK), Pushti Kormi (PK), Shasthya Shebika (SS) and Pushti Shebika (PS).* The identified activities were validated through review of the home fortification programme-related documents, such as job-aid of field-level staff (e.g. SK, PK, SS and PS) and monthly performance report.

The inputs generally used in the home fortification interventions were staff time, the home fortification materials, Pushtikona (a brand name of BRAC MNP) selling incentives, logistics and supply, monitoring, capital items, rental cost for contractual work relating to items with external organisations etc. We considered different inputs to calculate the cost of different activities as required (Table 1). For face-to-face counselling, inputs were: staff time of SS, PS, SK and PK, logistics and supply, monitoring done by SK/PK, PO and UM and incentives earned by SS/PS and SK/PK. For the social mobilisation activities (forum/advocacy session), the inputs were: staff time (of PO and UM), logistics and supply, materials (pen, notebook, brochure and file), refreshment, capital items (table, chair, fan, etc.) and rent of the venue. For mass media, the inputs were: popular theatre team (contracted out), banner, poster, technician, leaflets and invitation cost to the mass media session. These cost ingredients were classified into contract-out cost, staff cost, office space cost, transportation cost and capital cost considering the available structure of cost data.

**Table 1:** Sources of data for cost and effect estimation of Maternal, Infant and Young Child Nutrition home fortification programme

Data sources	Extracted information
Data collection from 7 selected upazilas: (a) Interview of staff (*n* 144) (b) Document review (e.g. expenditure document, incentive record, monthly performance report)	(a) Cost inputs (e.g. staff time, office rent, incentive cost) (b) Number of activities performed (e.g. number of home visit performed)
Programme record (e.g. under-five child register) for all 164 upazilas	Number of under-five children in the programme upazilas
Endline household survey (*n* 5237)	Coverage of the programme
	Prevalence of anaemia among compiler and non-compiler groups

All the activities of the home fortification programme were separated into start-up and implementation phases for the costing analysis^(24)^. The costs (capital and recurrent items) used to perform the activities until the roll-out of intervention was considered as the start-up cost of the home fortification programme^(24)^. Start-up cost was considered as a fixed capital item and annuitised over the 5-year programme period. The costs required to run the intervention in a typical post-start-up period when the programme is implemented are considered implementation costs^(24)^. The key activities of the programme and the required cost inputs are presented in Table 2.

**Table 2:** Key activities of the programme and required inputs

Activities	Inputs required
Start-up activities	
(1) Delivery channel and community mobilisation (training module, monthly performance report, Pustikona register, monitoring calendar and field operational guideline development; basic training of staff)	Staff time, printing, venue and refreshment
(2) Demand creation and message harmonisation (update communication and training materials, round table meeting on IYCF and research on consumer insights)	Staff time, printing, venue and refreshment
(3) Policy Enabling Environment and Programme Management (Stakeholder consultation; policy guideline, monitoring tools, evaluation framework development and management of programme activities)	Staff time, printing, venue, per diem and refreshment
Implementation activities	
(1) Household visit related (face-to-face counselling, demonstration of Pustikona use, selling of Pustikona and checking Pustikona card)	Staff time, transportation, incentive of staff, monitoring and materials
(2) Social mobilisation (forum/advocacy sessions at communities and mass media)	Staff time, banner, refreshment, printing, allowance, capital items used (table, chair, fan etc.) and contracted out costs
(3) Meeting on progress of activities (upazila-level meeting)	Staff time, venue, refreshment and transport
(4) Special refresher training (Training of SS and SK at upazila level)	Staff time, training materials, printing, refreshment, allowance and transportation
(5) Monitoring and overall programme management (head office-level meeting, training, printing of communication materials, stakeholder engagement and programme management)	Staff time, office rent, materials printing, contracted-out cost, refreshment, venue and per diem

IYCF, Infant and Young Child Feeding; SS, Shasthya Shebika; SK, Shasthya Kormi.

Estimation of cost from the programme perspective included all the costs incurred for the programme implementers. The user perspective cost included both direct (e.g. transportation cost to attend a forum and purchase of MNP) and indirect costs (e.g. time devoted during the forum by parents)^(25)^ incurred for receiving the services.

#### Effect estimation

For economic evaluation of the home fortification programme, the key outcome indicators were the total number of anaemia cases averted and the total number of DALY averted throughout the programme implementation period. Due to the unavailability of any comparison group in the evaluation design, the total number of anaemia cases averted was estimated using the change in anaemia prevalence considering only the endline survey between the complier group (ever taken MNP) and non-compiler groups (never taken MNP) of targeted 6–59 months aged children in 164 upazilas. This was possible as the endline sample was drawn considering the number of households having children aged 6–59 months rather than beneficiaries only. We used the estimated difference in the prevalence of anaemia between compiler and non-compiler groups as a crude effect of the programme, i.e. the number of anaemia case averted over the 5-year programme period, i.e. 2014–2018.

Total number of DALY averted from the home fortification programme was estimated by comparing home fortification programme outcomes with no intervention scenario^(26)^. Total DALY were estimated using the years of life lived with anaemia (YLD) and life years lost from mortality (YLL) among under-five children, using the following standard formula:
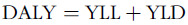



The YLD is the product of the prevalence rate, average years until remission of disease or death and the disability weight^(27)^. The YLL is estimated by multiplying the number of deaths by the standard life expectancy (from country-specific life table of WHO) at the age at which death occurs^(28)^.

Thus, YLD = Disease prevalence × average years until remission of disease or death × disability weight; YLL = Number of deaths × standard life expectancy at the age of death in years.

Finally, age-weighting and discounting of DALY estimates were done using 3 % discounting rate following the WHO guideline^(29)^. The YLD was estimated by using the prevalence rate of anaemia by severity level, under-five population in the programme area and disability weights. First, we estimated that around 5 358 604 under-five children were enrolled in the BRAC’s home fortification programme over the 5-year period considering crude birth rate of 19·4 per 1000 population, under-five mortality 53 per 1000 population, and population growth rate of 1·34 % (non-duplication in counting children over the 5 years)^(30)^. Second, we used the crude difference of anaemia prevalence among the compilers and non-compilers by the severity level at endline period to estimate the number of cases averted (shown in the result). Finally, disability weights were considered: 0·004 for mild anaemia cases, 0·052 for moderate anaemia cases and 0·149 for severe anaemia cases^(31)^.

Phiri *et al.* 2008 estimated 6·4 % mortality rate among the hospitalised cases of severe anaemia^(33)^. The number of anaemia-associated deaths in each scenario (home fortification programme and no intervention) was estimated using this rate and prevalence of severe anaemia. The disability weight for YLL estimation for the anaemia-related deaths was 1·00^(32)^. We used a template provided by the WHO for DALY calculation^(33)^. In the absence of a control group, the difference between the DALY estimates of home fortification programme and no intervention scenario was considered as the DALY averted due to the programme.

#### Estimation of incremental cost-effectiveness ratio

Cost per anaemia case averted and cost per DALY averted were estimated by dividing the programme cost with the number of anaemia case averted and number of DALY averted separately in the implementation area in comparison with a no intervention (do nothing) situation. As the programme was not implemented on the top of an existing intervention, the total implementation cost was considered as the additional costs due to the intervention. Further, in the absence of a comparison group, we considered the non-compiler group as comparison for measuring the effect of the programme assuming do nothing situation (zero cost and zero benefits) for measuring incremental cost-effectiveness ratio.

Considering the key programmatic factors that influenced the reduction of anaemia cases in the programme areas, contact coverage of MNP (if the child ever consumed food with MNP) and consumption of ≥30 MNP sachets have been considered in costing analysis. These measurements (cost per anaemia case averted and DALY averted) were calculated separately for the consumption of ≥30 MNP sachets (effective coverage) by the under-five children, using a similar approach.

#### Cost-effectiveness threshold

For reporting the cost-effectiveness of the home fortification programme, we used the threshold level proposed by the WHO. According to the WHO, an intervention is considered cost-effective if cost per DALY averted is <3 times national annual per capita GDP. If the costs per DALY averted is less than the national annual per capita GDP, the programme is considered highly cost-effective^(18,19)^.

#### Sensitivity analysis

A one-way sensitivity analysis was conducted to evaluate the changes in cost per DALY averted estimate due to uncertainty of the parameters. In the sensitivity analysis, we used the low and high value of selected parameters compared with their base values. Different values for discounting rate (0 % and 5 %)^(34)^, MIYCN home fortification programme coverage (5 % change), head office-level costs (10 % change) and unit costs of home visit-related activities (20 % change for each activities), i.e. face-to-face counselling, home visit for Pustikona-5 selling, checking calendar and demonstration of Pustikona-5, were used for sensitivity analysis. We assumed 10 % change in head office-level costs and 20 % change in the costs of all other home visit-related activities to observe their effect on cost per DALY averted estimate.

#### Data analysis

MS Excel and STATA (version 13) were used for data analysis in this study. The unit cost of activities at the community level (e.g. home visit for counselling) was estimated by valuing the resources utilised to perform the activities in seven selected upazilas. This unit cost was multiplied by average number of activities performed (extracted from Monthly Performance Reports of BRAC) per upazila and number of implementations upazilas over the programme period to estimate the total cost. Similarly, the unit cost of community mobilisation/advocacy session, monthly meeting and refresher training was estimated and multiplied by the number of activities performed per upazila per year. Upazila office-level maintenance cost was estimated separately considering the level of utilisation of shared cost items (e.g. office space and furniture) and 1-year equivalent cost of capital items^(25)^. Total costs of interventions, unit costs and distribution of costs across input categories were calculated, and the cost drivers were identified. Start-up cost, upazila-level implementation cost and head office-level cost were considered as the total intervention costs of the home fortification programme. Capital items were subject to discounting at 3 % over their useful lifetime, and all costs were adjusted for inflation. Exchange rate (1 USD = 78·8 BDT) in the fiscal year 2016 was used for expressing cost in US dollar (USD) equivalence of Bangladeshi Taka (BDT)^(30)^. Total number of anaemia case averted and total DALY averted was estimated as the effect of the programme.

#### Assumptions

To estimate the cost of the home fortification programme activities, some assumptions have been made. First, only the field-level implementation costs were included as the implementation costs of the programme. It was decided that the year 2016 was a good representation of the total implementation costs of a full year as the programme had been in full operation during that period. Based on the estimated number of activities performed in 2016, we assumed that equal numbers of home fortification activities were performed in all upazilas per year over the programme period. Considering the programme roll-out over the implementation period, we estimated cost of sixty-eight upazilas in the first year, 120 upazilas in the second year and all 164 upazilas in the following two and half years.

## Results

### Cost in start-up phase

The start-up cost of five different activities under ‘Delivery Channels and Community Mobilization’ component was 12·13 million BDT (0·16 million USD); cost of four activities under demand creation and message harmonisation was 4·13 million BDT (0·05 million USD), and cost of three activities under ‘Policy-enabling Environment and Programme Management’ was 19·21 million BDT (0·25 million USD). Thus, the total start-up cost was estimated to be 35·46 million BDT (0·46 million USD) (Table 3). The annualised start-up cost was estimated to be 7 709 775 BDT or 97 914 USD.

**Table 3:** Start-up costs of the Maternal, Infant and Young Child Nutrition home fortification programme

Delivery channels and community mobilisation	Cost (BDT)	Cost (USD)	% Share
Training module and monthly performance report development by implementers	521 254	6704	34·21
Training delivery	11 496 631	147 867	
Pushtikona register development	32 727	421	
Self-monitoring calendar development for mothers	43 249	556	
Field operation guideline development	31 105	400	
Sub-total in			
Thousands	12 133	156·1	
Million	12·13	0·16	
Demand creation and message harmonisation			
Review and updating of the existing training and IPC materials currently being used	1 456 453	18 733	11·63
Participation in roundtable on infant-feeding practices	15 653	201	
Conduct research on consumer insight and communication channel access mapping at the initial phase	2 653 648	34 131	
Sub-total			
Thousands	4126	53	
Million	4·13	0·05	
Policy-enabling environment and programme management			
Generate policy-enabling guidelines and political support for home fortification	6 939 110	89 249	54·16
Demand creation through focused ethnographic study and BCC working group	893 970	11 498	
Developing monitoring and evaluation tool aligned with the evaluation framework	11 373 270	146 280	
Sub-total in thousands (million)			
Thousands	19 206	247	
Million	19·21	0·25	
Total cost in thousands (million)			100
Thousands	35 465	456	
Million	35·46	0·46	
Annual equivalent start-up cost	7 709 755	97 840	
Total annualised start-up cost (5-year programme period)	38 548 775	489 200	

BCC, behaviour change communication; IPC, Interpersonal Communication; USD, US dollar.

### Implementation cost per upazila per year

The average implementation costs per upazila per year of the home fortification community programme are presented in Table 4. The highest cost was observed for the home visit-related activities 1·2 million BDT or 15·5 thousand USD (71·45 % of total). The average unit cost for home visits related four activities was estimated as providing home fortification counselling to mothers (10·4 BDT; 0·13 USD), face-to-face counselling regarding Pushtikona selling (8·8 BDT; 0·11 USD), demonstration of Pushtikona (12·4 BDT; 0·16 USD) and checking Pushtikona calendar (3·2 BDT; 0·04 USD) (see online supplementary material, Supplemental Table 2). The per-visit demonstration-related cost was the highest because each demonstration process required more time than home visit-related activities. The average cost for home fortification-related advocacy sessions was estimated to be 10 464 BDT (529 BDT; 6·7 USD per participant). Participant’s allowance and refreshment were the two major cost drivers (more than 50 %) for such advocacy sessions (see online supplementary material, Supplemental Table 3). On an average 16, such advocacy session took place per upazila in a year that corresponds to 9·8 % of the implementation costs. The cost per year for the MIYCN upazila health office was estimated at 181 806 BDT (2307 USD) which was 10·7 % of the implementation cost (see online supplementary material, Supplemental Table 4). The cost per monthly meeting and special refresher training was estimated to be 8602 BDT and 5775 BDT, respectively. In a year on an average, 12 monthly meeting and 6 special training were conducted in one upazila that corresponds to 6·05 % and 2·03 % of the yearly implementation cost.

**Table 4:** Yearly implementation cost per upazila/sub-district

Cost component	Cost per activity (BDT)	Average activities performed in a year	Estimated cost in BDT	Estimated cost in USD	% share of costs
Home visit					
Face-to-face counselling	10·4	53 430	555 672	7049·89	32·57
Demonstration of the use of Pustikona-5	12·4	4013	49 761	631·33	2·92
Home visit for Pustikona-5 selling	8·8	50 762	446 704	5667·39	26·18
Checking Pustikona-5 calendar/card	3·2	52 156	166 899	2117·47	9·78
Subtotal			1 219 036	15 466·00	71·45
Social mobilisation/advocacy sessions	10 464·0	16	167 424	2124·13	9·81
Maintaining upazila office (e.g. office rent, utilities and furniture)	–	1	181 807	2306·61	10·66
Monthly meeting	8602·0	12	103 219	1309·56	6·05
Special refresher training	5775·0	6	34 651	439·62	2·03
Subtotal			487 101	6179·92	28·55
Grand total			1 706 138	21 646·00	100·00

USD, US dollar.

* 1 USD is equivalent to 78·8 BDT.

#### Total implementation cost of the home fortification programme

Table 5 presents the total implementation costs of the home fortification programme at 164 upazilas of Bangladesh. The highest cost for implementation was associated with the activities related to home visits (708·40 million BDT or 9·00 million USD). Social mobilisation, monthly meetings, special refresher training and upazila office maintenance cost were estimated to be 283·06 million BDT or 3·59 million USD. The head office-level cost in the implementation period was estimated to be 120·16 million BDT or 1·52 million USD. Thus, the total implementation cost was estimated to be 1111·63 million BDT or 14·12 million USD. The annualised start-up cost for 5 years and implementation cost constituted the total home fortification programme cost which was estimated to be 1150·18 million BDT or 14·6 million USD. The estimated cost borne by the home fortification programme was 896·15 million BDT or 11·38 million USD. This excludes the opportunity cost for resources used in the home fortification programme, e.g. voluntary involvement of CHWs’ time, excluding the incentive. Additionally, the implementation cost does not include cost for overall programme monitoring and management during this phase.

**Table 5:** Total implementation cost of the Maternal, Infant and Young Child Nutrition home fortification programme in all 164 upazilas

Cost component	Cost per activity (BDT) (Col. A)	Total activities performed in the programme period (Col. B)*	Estimated cost in million BDT (without discounting) (A × B)	Estimated cost in million BDT (after discounting)	Estimated cost in thousands USD	Estimated cost in million USD†
Home visit						
Face-to-face counselling	10·4	31 951 140	332·29	322·91	4101	4·10
Demonstration of the use of Pustikona-5	12·4	2 399 774	29·76	28·92	367	0·37
Home visit for Pustikona-5 selling purpose	8·8	31 189 288	267·13	259·59	3297	3·30
Checking Pustikona-5 calendar/card	3·2	30 355 676	99·81	96·99	1232	1·23
Subtotal			728·98	708·40	8997	9·00
Social mobilisation/advocacy sessions	10 464·0	9568	100·12	97·29	1236	1·24
Maintaining upazila office	–		108·72	105·65	1342	1·34
Monthly meeting	8602·0	7176	61·73	59·98	762	0·76
Special refresher training	5775·0	3588	20·72	20·14	256	0·26
Subtotal			291·29	283·06	3595	3·59
Total implementation cost at field level			1020·27	991·5	12 592	12·6
Total cost at head office level (BRAC and SMC)			122·40	120·16	1526	1·53
Total implementation cost	–	–	1142·67	1111·63	14 118	14·12
Cost not borne by the programme (e.g. voluntary staff)			221·49	215·48	2737	2·74
Cost borne by the programme (direct cost)			921·17	896·15	11 381	11·38
Total annualised start-up costs			38·55	38·55	489	0·49
Total programme costs (start-up and implementation)			1181·22	1150·18	14 607	14·6
User cost of the programme			467·43	467·43‡	5932	5·93
Total programme cost considering user cost			1648·65	1617·61	20 539	20·53

USD, US dollar.

* Annual number of activities (presented in Table 3) was multiplied by 598 (68 upazilas in the first year, 120 upazilas in the second year and 164 upazilas in the following two and half years).

† 1 USD is equivalent to 78·8 BDT.

‡ Not discounted.

The user cost of the home fortification programme was estimated to be 467·43 million BDT (5·93 million USD) (see online supplementary material, Supplemental Table 5). Considering the user costs, the total programme costs stand at 1617·61 million BDT (20·53 million USD).

### Estimated outcome from the intervention

The prevalence of anaemia was estimated to be 39·6 % (*n* 1658) among the under-five children who did not consume any MNP during the programme period (non-compilers) and 27·3 % (*n* 2104) among that of consumed any amount of MNP (compilers) in the endline period. The average impact of the home fortification programme on the reduction of anaemia among the under-five children was estimated to be 12·3 % points. This corresponds to the aversion of 657 778 under-five anaemia cases in the programme implementation period of the 5 358 604 exposed population (Table 6).

**Table 6:** Number of anaemia cases, deaths and estimated disability-adjusted life years averted from Maternal, Infant and Young Child Nutrition home fortification programme

Indicator	No sachet	Any sachet	Average impact (% change)	Number of anaemia cases/deaths averted	DALY averted
Overall anaemia	39·6 %	27·3 %	12·3 %	657 778	91 588
For 30 or more sachets consumption	39·6 %	24·5 %	15·1 %	367 172	55 786
Anaemia by severity					
Severe	0·18 %	0·14 %	0·04 %	2049	5607
Moderate	17·07 %	10·65 %	6·42 %	344 104	80 520
Mild	22·32 %	16·50 %	0·81 %	311 486	1374
Deaths				140	4087

While considering the level of severity, the prevalence of severe anaemia was reduced from 0·18 % to 0·14 %, moderate anaemia from 17·06 % to 10·65 % and mild anaemia from 22·32 % to 16·50 % between non-compiler and compiler groups. The average effect of the programme in the change of anaemia prevalence in terms of severity level was estimated at 0·04 % of severe cases, 6·42 % of moderate cases and 5·81 % of mild cases. The estimated 140 deaths were averted through the home fortification programme. Considering the corresponding DALY weight as per severity level, estimated 91 588 DALY were averted from the home fortification programme. The average effect of consuming thirty or more sachets of MNP on anaemia reduction was higher (15·1 % *v*. 12·3 %) compared with the overall consumption. It was estimated that 55 786 DALY were averted among the children who consumed thirty or more sachets of MNP in the home fortification programme period.

### Estimated incremental cost-effectiveness ratio

Cost per anaemia case averted was estimated to be 1749 BDT (22·2 USD). However, the cost per DALY averted was estimated to be 12 558 BDT (159·3 USD). Cost per anaemia case averted and DALY averted while consuming thirty or more sachets of MNP was estimated to be 1653 BDT (21·0 USD) and 10 877 BDT (137·9 USD), respectively. Considering the user costs in the programme, the cost per anaemia case averted was estimated to be 2459 BDT (31·2 USD) and cost per DALY averted was estimated to be 17 662 BDT (224·0 USD) (Table 7).

**Table 7:** Cost-effectiveness ratio for overall effect of the Maternal, Infant and Young Child Nutrition home fortification programme

Nutritional indicator	Number of anaemia cases or DALY averted	Associated cost (million BDT)	Cost per anaemia case or DALY averted (BDT)	Cost per anaemia case or DALY averted (USD)
Anaemia cases averted	657 778	1150·18	1749	22·2
Overall DALY averted	91 588	1150·18	12 558	159·3
Cost:outcome ratio for 30+ sachet consumption				
Anaemia cases averted	367 172	606·78	1653	21·0
DALY averted	55 786	606·78	10 877	137·9
Cost:outcome ratio, including user cost				
Anaemia cases averted	657 778	1617·61	2459	31·2
Overall DALY averted	91 588	1617·61	17 662	224·0

DALY, disability-adjusted life years.

Considering the WHO-provided GDP threshold, a programme is very cost-effective if the cost per DALY averted is less than one time of GDP per capita. Since the cost per DALY averted is 159·3 USD for the home fortification programme and it is less than one time of GDP per capita (1516·5 USD), this programme is very cost-effective.

#### Findings from sensitivity analysis

**Fig. 1:**
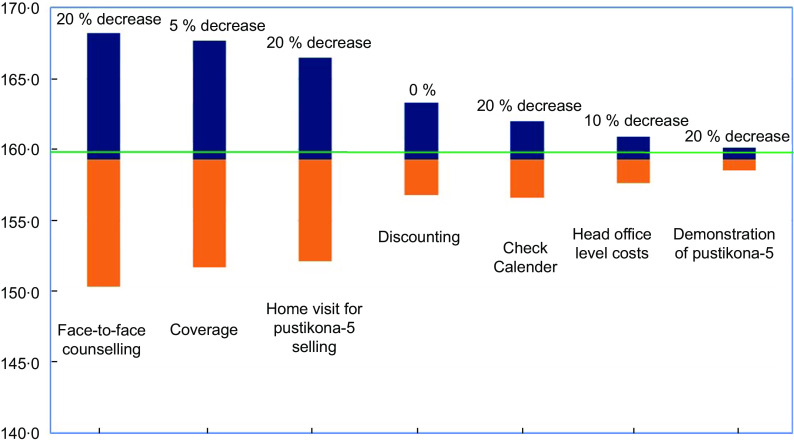
Sensitivity analysis showing the impact of changes in parameters on the cost per DALY averted. 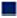, High; 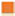, low

The result from sensitivity analysis showed that MIYCN home fortification programme coverage and costs for face-to-face counselling were the two important parameters that influenced the cost per DALY averted estimate (Fig. 1). Face-to-face counselling was the most influential parameter as 20 % increase in counselling, cost per DALY averted increased by 5·6 % (168·2 USD). If the programme coverage was increased by 5 % the costs per DALY averted reduced to 151·7 USD and if it was decreased by 5 % the costs increased to 167·7 USD. The cost per DALY averted estimate ranged between 156·8 USD and 163·3 USD, while 0–5 % discount rate was applied, respectively, in the calculation. The programme was still cost-effective (cost per DALY averted less than GDP per capita), while we considered uncertainty of the parameters in the sensitivity analysis.

## Discussion

The programme had an estimated total start-up cost of 35·46 million BDT (460 000 USD), and the total implementation cost for the project period was 1111·63 million BDT (14·12 million USD). In the start-up phase, most of the costs were incurred during the training of CHW, which accounted for 34·2 % of the total start-up costs. Home visit-related activities accounted for the major costs incurred during the implementation phase 71·45 % of the total implementation costs per year. The cost per anaemia case and per DALY averted from the BRAC’s home fortification programme in Bangladesh was estimated to be 22·2 USD and 159·3 USD, respectively. Considering outcomes as anaemia cases averted and DALY averted among the under-five children, the home fortification with MNP programme was very cost-effective. The cost per anaemia case averted and DALY averted was comparatively lower among the under-five children who consumed thirty or more sachets of MNP during the programme period meaning that such consumption of MNP was more cost-effective intervention from programme perspective compared with the overall consumption. In both start-up and implementation phases of the intervention, personnel costs accounted for a large share of the MIYCN home fortification programme costs. The programme coverage and costs for face-to-face counselling were influential parameters for cost per DALY averted estimate in the one-way sensitivity analysis. However, the programme was still cost-effective considering the uncertainty of the parameters.

Our cost-effectiveness estimates in terms of measured outcomes are consistent with other studies, although there are significant variations in cost estimates of micronutrient interventions, and the sources of these variations can be numerous^(35)^. A study conducted in China on micronutrient supplementation intervention found that the cost per DALY averted was 179 USD, and the programme was very cost-effective^(36)^. Another study in Peru found that an investment of 1·51 USD for the consumption of multi-micronutrients for each community member resulted in 1 % prevention of anaemia per community member^(37)^. The social entrepreneurship model implemented by Living Goods and BRAC Uganda led to a 27 % reduction in under-five child mortality at an estimated average cost of 71 USD per life-year gained^(38)^. Unlike the MIYCN home fortification programme in Bangladesh, the CHW-driven social entrepreneurship model in Uganda delivered a wide range of products (e.g. mosquito nets, water purification tablets, vitamins, ORS, Zn, antimalarial drugs, pampers, soap and toothpaste) and therefore achieve impacts through multiple, simultaneous, pathways. Further, Björkman *et al.* 2017 estimated reduction in under-five mortality (any cause) and quality-adjusted life years gained as effectiveness, while the scope of this study was to estimate the effect on anaemia aversion only. Since the social entrepreneurship model in Uganda delivered a wide range of products through CHW, the programme may have the benefits of economy of scale (i.e. saving in costs by an increased level of production) which resulted in less cost per life saved compared with the home fortification programme (159·3 USD). Moreover, the embedded process evaluation and a need for careful documentation to produce lessons learned might have increased the implementation costs more than regular implementation. As such, several studies of different delivery models and bundled interventions report a variety of cost-effectiveness impact of home fortification with MNP.

Documenting and reporting costs of any programme have several benefits, including monitoring the performance of service delivery, setting efficiency targets or ascertaining the attainment of benchmarking of services across sectors, and considering or making a case for investment decisions. Cost estimation is a prerequisite for scaling up decisions and assessing sustainability of a programme. However, most studies conducted in Bangladesh used a top-down less detailed approach for cost analysis of health programme^(17)^. The activity-based ingredient costing approach is a much detailed costing approach than the top-down approach and allows better understanding and managing total cost^(23)^. Results from this costing exercise are particularly useful for planning budgets and financing for the initiation of the home fortification programme in a new area, in addition to rolling out this cost-effective programme nationally. Additionally, by analysing the impact of different home fortification activities, cost containment can be practised in the ongoing programme and in future programmes. As indicated earlier, separating the total costs into those at start-up and implementation phases were effective for utilising the results of this study for planning, budgeting and financing as well as the cost management of future home fortification programmes.

One limitation of this study is that we could not capture some costs for partner coordination and policy-level activities at the head office level. These included programmatic analysis, policy sensitisation and strategic management of the MIYCN consortium. However, core costs for monitoring were captured for field-level implementation. We assumed that exclusion of the cost for such activities in the implementation phase will not affect the preparation of a financial plan for scaling-up of the home fortification programme especially since such activities will be performed by the existing management body of the health system in LMIC. The training and sensitisation activities required for strengthening the existing management body for effectively implementing this programme were captured in the cost analysis of the start-up phase. WHO guide to cost-effectiveness analysis allows excluding some of the costs of central administration that are part of the overall management and unrelated to the development and implementation of the health interventions since such activities may be done in the country for the available resources^(24,29)^. However, we ensured that the partner coordination and policy-level activities borne during the start-up period were included due to the major role in designing the programme and sensitising it at the health system level. Therefore, the unit cost at the start-up period was not underestimated.

The home fortification programme under assessment may have a long-term effect on demand of MNP after completion of programme implementation in the upazilas. This may play a role in averting anaemia case in those upzailas after programme implementation. We were unable to consider this effect in the cost-effectiveness estimation because of the absence of data. However, the cost-efficiency of the programme might be further increased if this additional effect could be considered in the calculation. It should be noted here, if a manufacturing firm pushes the micronutrient powders into other upazilas without the support of a programme similar to MIYCN which offered face-to-face counselling on benefits of the powder and demonstrate its use, the effect on anaemia can be lower. Therefore, this analysis is presenting an upper bound of the effects, and the cost per beneficiary could be higher. Future studies can be conducted to assess the effect of micronutrient powders on anaemia in the absence of programmatic support.

We did not find any estimate on the proportion of under-five deaths due to anaemia in Bangladesh context through literature search. Therefore, for estimating YLL, we used the fatality rate for severe anaemia estimated by Phiri *et al.* 2008 in Malawi context as a proxy estimate. However, there may be bias because of the contextual differences between Bangladesh and Malawi settings which is another potential limitation of this study^(32)^. This cost-effectiveness analysis focused on the economic viability of the MIYCN home fortification programme in the context of Bangladesh. However, further research is required to understand the affordability to scale up this programme nationally considering the fiscal space of the government and development partners to allocate more resources on micronutrients along with their other competing interest.

## Conclusions

Bangladesh, with its high undernutrition rates, is expected to invest in nutrition, in order to foster the economic growth, that will plausibly occur consequent to improvements in nutritional status. Findings from the current study suggest that home fortification with MNP through the MIYCN home fortification programme was very cost-effective in averting the anaemia-associated health burden. This programme is a feasible mechanism for delivering cost-effective micronutrients to a large number of children in Bangladesh and similar low-income contexts. The programme generally influences the uptake of Pustikona-5 and, consequently, anaemic condition of children, which makes the investment worthy. The activity-based start-up and implementation cost estimates in this study will be useful for preparing a financial plan for scaling-up the home fortification programme in Bangladesh or initiating this programme in other similar low-income countries. Initiatives can be taken by the programme managers to better manage the cost-driving activities, identify efficiencies in delivery and reduce costs without influencing the impact of the programme.

## Supplementary material

For supplementary material accompanying this paper visit https://doi.org/10.1017/S1368980020003602. click here to view supplementary material
