# Supplementary material for: Cost-effectiveness of a market-based home fortification of food with micronutrient powder programme in Bangladesh
Source: Public Health Nutr. 2020 Oct 29;24(Suppl 1):s59–70. doi: 10.1017/S1368980020003602 (PMC8042576; doi:10.1017/S1368980020003602)
Supplement: Supplementary file 1 [file S1368980020003602sup001.zip › S1368980020003602sup005.docx]

**Supplementary table 1.** Number of field level staff interviewed in seven selected upazilas

| **Upazila** | **Number of field level staff interviewed** | | | | | | | |
| --- | --- | --- | --- | --- | --- | --- | --- | --- |
|  | **Shaystha Sebika** | **Pusti Sebika** | **Shaystha Kormi** | **Pusti Kormi** | **Branch Manager** | **Program organizer** | **Filed Officers** | **Total interview** |
| Beyanibazar | 6 | 6 | 3 | 3 | 1 | 0 | 3 | **22** |
| Shahajanpur | 6 | 6 | 3 | 3 | 1 | 0 | 1 | **20** |
| Gobindogonj | 10 | 0 | 5 | 0 | 1 | 0 | 1 | **17** |
| Laksham | 7 | 7 | 3 | 3 | 1 | 3 | 1 | **25** |
| Monirampur | 5 | 5 | 3 | 3 | 1 | 2 | 4 | **23** |
| Borguna Sador | 6 | 6 | 3 | 3 | 2 | 2 | 3 | **25** |
| Muktagacha | 5 | 0 | 5 | 0 | 1 | 0 | 1 | **12** |
| **Total** | **45** | **30** | **25** | **15** | **8** | **7** | **14** | **144** |
