# Supplementary material for: Cost-effectiveness of a market-based home fortification of food with micronutrient powder programme in Bangladesh
Source: Public Health Nutr. 2020 Oct 29;24(Suppl 1):s59–70. doi: 10.1017/S1368980020003602 (PMC8042576; doi:10.1017/S1368980020003602)
Supplement: Supplementary file 1 [file S1368980020003602sup001.zip › S1368980020003602sup001.doc]

| **Supplementary table 2. Cost for household visit activities** | | | | | |
| --- | --- | --- | --- | --- | --- |
| **Cost component** | | **face-to-face home-fortification counselling** | **Selling Pushtikona** | **Demonstration of the use Pushtikona** | **Checking Pushtikona calendar/card** |
| *Staff cost* | PS/SS | 1.4 | 0.6 | 1.8 | 0.3 |
| *Travel cost* | Time | 0.2 | 0.2 | 0.2 | 0.2 |
|  | Transportation | 0.1 | 0.1 | 0.1 | 0.1 |
| **Subtotal** |  | **1.7** | **0.8** | **2.0** | **0.6** |
| *Staff cost* | PK/SK | 4.7 | 3.9 | 5.6 | 1.0 |
| *Travel cost* | Time | 0.6 | 0.6 | 0.6 | 0.6 |
|  | Transportation | 0.3 | 0.3 | 0.3 | 0.3 |
| ***Subtotal*** |  | **5.6** | **4.8** | **6.5** | **1.9** |
| *Incentive* | | - | 1.5 | - | - |
| *Monitoring* | BM/UM | 1.9 | 1.2 | 2.4 | 0.5 |
|  | PO/FO | 1.2 | 0.5 | 1.5 | 0.3 |
| ***Subtotal*** |  | **3.1** | **1.7** | **3.9** | **0.7** |
| **Materials** | | - | - | 0.9 | 0.1 |
| Cost (not borne by the programme) | | 1.7 | 0.8 | 2.0 | 0.6 |
| Total Cost (borne by programme) | | 8.7 | 8.0 | 10.4 | 2.7 |
| ***Total cost per visit*** | | ***10.4*** | ***8.8*** | ***12.4*** | ***3.2*** |
| **USD*** |  | **0.13** | **0.11** | **0.16** | **0.04** |
| ***1 USD is equivalent to 78.8 BDT** | | | | | |
