# Supplementary material for: Cost-effectiveness of a market-based home fortification of food with micronutrient powder programme in Bangladesh
Source: Public Health Nutr. 2020 Oct 29;24(Suppl 1):s59–70. doi: 10.1017/S1368980020003602 (PMC8042576; doi:10.1017/S1368980020003602)
Supplement: Supplementary file 1 [file S1368980020003602sup001.zip › S1368980020003602sup003.doc]

| **Supplementary table 5.** User cost of the programme | | | |
| --- | --- | --- | --- |
| **Cost component** | **Cost per activities** | **Average number of activities done in 164 upazilas in five years/participants** | **Cost in BDT** |
| Face-to-face counselling | 4 | 31,951,140 | 134,194,788 |
| Demonstration of the use of Pustikona-5 | 3 | 2,399,774 | 6,239,412 |
| Checking Pustikona-5 calendar/card | 0.7 | 30,355,676 | 20,641,860 |
| Mothers advocacy session | 19.1 | 58,875 | 1,142,180 |
| Fathers advocacy session | 104.5 | 40,664 | 4,249,388 |
| Cost for MNP |  |  | 300,966,612 |
| **Total** | - |  | **467,434,241** |
