# Supplementary material for: Cost-effectiveness of a market-based home fortification of food with micronutrient powder programme in Bangladesh
Source: Public Health Nutr. 2020 Oct 29;24(Suppl 1):s59–70. doi: 10.1017/S1368980020003602 (PMC8042576; doi:10.1017/S1368980020003602)
Supplement: Supplementary file 1 [file S1368980020003602sup001.zip › S1368980020003602sup004.doc]

| **Supplementary table 4.** Health office cost per upazila per year | |  |
| --- | --- | --- |
| **Cost component** | **Amount (BDT)** | **USD*** |
| Capital cost | 42,141.98 | 534.66 |
| Floor space rent | 56,422.37 | 715.84 |
| Supplies | 14,470.71 | 183.59 |
| Stationeries | 31,610.71 | 401.05 |
| Utilities | 37,160.74 | 471.46 |
| **Total cost** | **18,1806.5** | **2306.60** |
| *1 USD=78.8 BDT | |  |
