# Supplementary material for: Cost-effectiveness of a market-based home fortification of food with micronutrient powder programme in Bangladesh
Source: Public Health Nutr. 2020 Oct 29;24(Suppl 1):s59–70. doi: 10.1017/S1368980020003602 (PMC8042576; doi:10.1017/S1368980020003602)
Supplement: Supplementary file 1 [file S1368980020003602sup001.zip › S1368980020003602sup002.doc]

| **Supplementary table 3.** Cost of forum/advocacy sessions | | | | | | | | | | |  | |
| --- | --- | --- | --- | --- | --- | --- | --- | --- | --- | --- | --- | --- |
| **Cost component** | **Input** | | **Father** | **Mother** | **Teacher** | **Village doctor** | **Elite person** | **Religious leader** | **Health Assistant** | **Community leader** | | ***Average cost per session (all session)*** |
| Fixed cost items | *Capital items* | | 69.63 | 58.32 | 61.28 | 89.82 | 42.62 | 61.25 | 41.12 | 79.04 | | *62.9* |
|  | Venue rent | | 35.71 | 31.24 | 29.62 | 74.67 | 49.21 | 47.19 | 74.68 | 36.16 | | *47.3* |
| ***Total fixed cost*** | | | **105.33** | **89.56** | **90.90** | **164.48** | **91.83** | **108.44** | **115.81** | **115.20** | | ***110.2*** |
| Variable cost items | *Materials* | | 1484.43 | 1704.83 | 1319.86 | 1365.29 | 1387.50 | 1269.00 | 230.00 | 1278.33 | | *1,254.9* |
|  | *Allowance* | *Facilitator* | 700.00 | 700.00 | 700.00 | 700.00 | 700.00 | 700.00 | 700.00 | 700.00 | | *700.0* |
|  |  | *Participants* | 4000.00 | 3933.33 | 3971.43 | 3942.86 | 4000.00 | 4000.00 | 4000.00 | 3800.00 | | *3,956.0* |
|  | *Personnel* | *UM* | 196.60 | 180.71 | 179.87 | 223.79 | 267.71 | 210.82 | 313.73 | 112.94 | | *210.8* |
|  |  | *PO* | 449.12 | 464.93 | 464.93 | 322.61 | 439.63 | 506.05 | 237.21 | 85.40 | | *371.2* |
|  |  | *FO* | 335.09 | 255.31 | 247.80 | 265.63 | 215.89 | 166.14 | - | 262.82 | | *218.6* |
|  |  | *PK /SK* | - | 62.22 | - | - | - | - | - | - | | *7.8* |
|  |  | *SS* | 11.36 | 32.39 | - | - | 22.16 | - | - | - | | *8.2* |
|  |  | *RM* | - | - | - | - | 411.93 | - | - | - | | *51.5* |
|  | *Refreshment* | *Facilitator* | 117.65 | 120.00 | 117.65 | 117.65 | 115.56 | 117.65 | 80.00 | 120.00 | | *113.3* |
|  |  | *Staff* | 334.12 | 435.00 | 280.00 | 369.41 | 408.89 | 225.88 | 160.00 | 315.00 | | *316.0* |
|  |  | *Participants* | 2352.94 | 2355.00 | 2338.82 | 2317.65 | 2311.11 | 2352.94 | 1600.00 | 2295.00 | | *2,240.4* |
|  | *Travel for invitation* | *FO* | 176.98 | 206.86 | 166.27 | 216.69 | 135.14 | 214.14 | - | 232.65 | | *168.6* |
|  |  | *PO* | 407.43 | 199.26 | 609.91 | 234.45 | 214.60 | 215.31 | 351.28 | - | | *279.0* |
|  | *Arrangement Items* | *Banner* | 463.57 | 474.17 | 463.57 | 463.57 | 450.00 | 463.57 | 400.00 | 481.67 | | *457.5* |
| ***Total variable cost*** | |  | **11,029.29** | **11,124.01** | **10,860.11** | **10,539.59** | **11,080.11** | **10,441.51** | **8,072.22** | **9,683.81** | | ***1,353.8*** |
| **Total cost per session** | |  | **11,134.62** | **11,213.57** | **10,951.02** | **10,704.07** | **11,171.94** | **10,549.95** | **8,188.02** | **9,799.01** | | ***10,464.0*** |
| **Average number of participants** | | | ***20*** | ***19.67*** | ***19.86*** | ***19.71*** | ***20*** | ***20*** | ***20*** | ***19*** | | ***19.8*** |
| **Cost per participant** | |  | ***556.73*** | ***570.08*** | ***551.41*** | ***543.08*** | ***558.60*** | ***527.50*** | ***409.40*** | ***515.74*** | | ***529.1*** |
| **(USD* value)** | | | ***7.06*** | ***7.23*** | ***7.00*** | ***6.89*** | ***7.09*** | ***6.69*** | ***5.19*** | ***6.54*** | | ***6.71*** |
| *1 USD=78.8 BDT as of 7 December 2016 | | | | | | | | | | |  | |
